# Eco-Friendly NiO/Polydopamine Nanocomposite for Efficient Removal of Dyes from Wastewater

**DOI:** 10.3390/nano12071103

**Published:** 2022-03-27

**Authors:** Marwa A. El-Ghobashy, Hisham Hashim, Moustafa A. Darwish, Mayeen Uddin Khandaker, Abdelmoneim Sulieman, Nissren Tamam, Sergei V. Trukhanov, Alex V. Trukhanov, Mohamed A. Salem

**Affiliations:** 1 Chemistry Department, Faculty of Science, Tanta University, Al-Geish St., Tanta 31527, Egypt; masalem@science.tanta.edu.eg; 2 Physics Department, Faculty of Science, Tanta University, Al-Geish St., Tanta 31527, Egypt; hh@science.tanta.edu.eg (H.H.); mostafa_ph@science.tanta.edu.eg (M.A.D.); 3 Centre for Applied Physics and Radiation Technologies, School of Engineering and Technology, Sunway University, Petaling Jaya 47500, Malaysia; mayeenk@sunway.edu.my; 4 Department of Radiology and Medical Imaging, Prince Sattam Bin Abdulaziz University, P.O. Box 422, Alkharj 11942, Saudi Arabia; a.sulieman@psau.edu.sa; 5 Department of Physics, College of Science, Princess Nourah Bint Abdulrahman University, P.O. Box 84428, Riyadh 11671, Saudi Arabia; nmtamam@pnu.edu.sa; 6 Laboratory of Magnetic Films Physics, SSPA “Scientific and Practical Materials Research Centre of NAS of Belarus”, 19, P. Brovki Str., 220072 Minsk, Belarus; truhanov86@mail.ru; 7 Laboratory of Single Crystal Growth, South Ural State University, 76, Lenin Av., 454080 Chelyabinsk, Russia

**Keywords:** polydopamine, nanocomposites, removal, dyes, NiO, adsorption

## Abstract

The rapid development of industries discharges huge amounts of wastewater that contain surface water. For this reason, we used NiO/polydopamine (NiO/PDA) nanocomposite as an efficient material for the removal of Methyl violet 2B from water. It was synthesized and then characterized by Fourier Transform Infrared (FT-IR) spectroscopy, X-ray Diffraction (XRD), Scanning Electron Microscopy (SEM), Energy Dispersive X-ray (EDX) analysis, Transmission Electron Microscopy (TEM), and Brunauer–Emmett–Teller (BET). The EDX analysis confirmed the presence of O, Ni, N, and C. The composite has an average particle size of 18 nm. Its surface area is 110.591 m^2^/g. It was found that the efficiency of dye removal by adsorption on NiO/PDA exceeded that of bare NiO. The adsorption capacity of NiO and NiO/PDA are 126 and 284 mg/g, respectively. The effects of adsorbent dose, dye concentration, and pH on the removal efficiency were examined. The efficiency increased with increasing the adsorbent dose and pH, but dropped from 85 to 73% within 30 min as the initial dye concentration was increased from 0.984 to 4.92 mg/L. Such a drop in the removal efficiency is due to the blocking of the surface-active sites of NiO/PDA, with the high population of dye molecules derived from the continuous increase in dye concentration. The adsorption results of the dye fitted well with the pseudo-second-order kinetics and Langmuir isotherm. The reusability data showed that NiO/PDA was stable across three adsorption–regeneration cycles, thus it can be considered a good recyclable and efficient adsorbent. Because of these results, it can be considered that this method can be applied for the treatment of wastewater.

## 1. Introduction

The rapid development of technology in different fields is currently considered a double-edged sword. Dyes have many industrial applications; they are used in textiles, papers, plastics, leathers, food, and cosmetics, and in most cases, a large amount of industrial wastewater is discharged, which is harmful to aquatic life and humans. Many azo dyes and their intermediates have toxic effects on human health because of their carcinogenicity [1]. Dyes usually have complex aromatic molecular structures that render them difficult to biodegrade.

Many treatment methods have been applied to purify wastewater that is contaminated with dyes. These include chemical coagulation/flocculation [2], ozonation [3], oxidation [4], photodegradation [5], and aerobic or anaerobic treatment [6]. Each technique suffers from some limitations such as the design, separation efficiency, and total cost. Therefore, adsorption has been considered a good technique because it shows a high adsorption capacity for the uptake of dyes and heavy metals. It is particularly competitive, cost-effective, and efficient. Normally, the adsorption efficiency is affected by the physical and chemical properties of both the adsorbent and adsorbate. The selectivity of an adsorbent is based on its capacity, surface area, availability, and total cost. For these purposes, porous metal oxides have received attention because of their large surface area and high capacity for adsorption [7].

Nickel(II) oxide (NiO) has significant advantages for environmental compatibility and high thermal and chemical stability. It has widely been used in lithium-ion batteries [8], gas sensors [9], supercapacitors [10], and catalysts [11]. Zheng et al. [12] used NiO microspheres as an adsorbent for the removal of Congo Red. Heavy metal ions were also removed by NiO [13]. Shehadeh et al. [14] used the modified nickel oxide nanoparticles with diatomite for the adsorption of Basic Red 46. The NiO nanoparticles prepared by a green hydrothermal method and NiO nanobelts synthesized hydrothermally were utilized for the removal of dyes [15,16]_ENREF_15_ENREF_15. The modified NiO with sepiolite and Fe_3_O_4_ to produce NiO/sepiolite and Fe_3_O_4_/NiO nanocomposites of high adsorption capacities were also applied for the removal of Congo Red from wastewater [17,18].

Polydopamine (PDA) is an eco-friendly biopolymer. It has applications in the biomedical areas and environmental science as a catalyst carrier for organic pollutant decomposition [19], and is an active adsorbent for the purification of water [20]. It has been applied in energy storage, water splitting, and various engineering fields [21]. PDA is an insoluble biopolymer and a dark brown material produced by the oxidative polymerization of dopamine (DA). It is a synthetic form of melanin (eumelanin) that is naturally occurring [22,23,24]. Recently, polydopamine was modified with some materials and used in the wastewater treatment field. The polydopamine/Ag microspheres were applied for the removal of Methylene Blue from the solution [25]. Further, the use of intercalated nanochannels of graphene oxide membranes with polydopamine (PDA/HGO) recovered the dye efficiently [26]. The modified polydopamine with water-soluble polyethylenimine that grafted into the framework of melamine foam and applied in the purification of wastewater displayed high adsorption capacity for Cr (VI) [27].

According to the advantages of NiO and PDA, we report in this paper the modification of NiO nanoparticles with polydopamine to form the NiO/PDA nanocomposite, which was synthesized and used for the first time for the efficient removal of Methyl Violet 2B (MV2B) from wastewater. 

## 2. Materials and Methods

### 2.1. Materials

**Figure 1:**
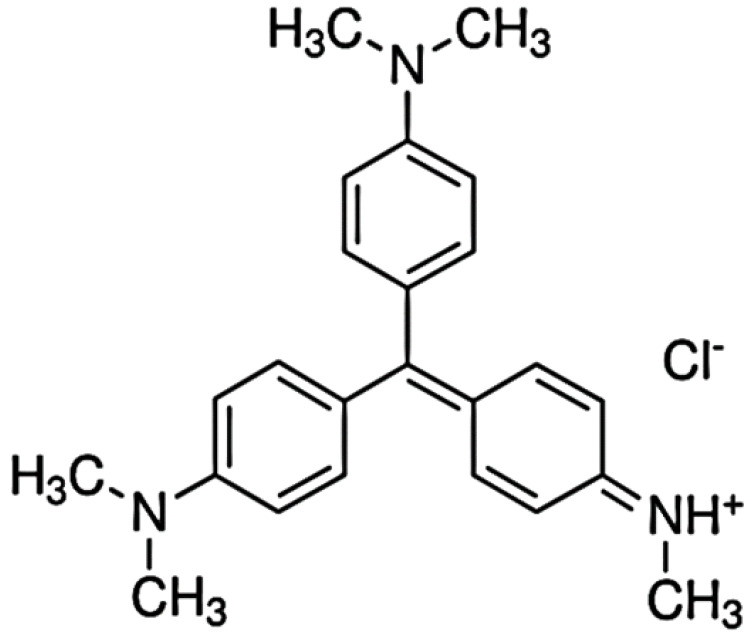
Structure of Methyl Violet 2B (MV2B).

Methyl Violet 2B (MV2B) (Figure 1) and Chromotrope 2B were supplied by Sigma-Aldrich (St. Louis, MO, USA). Oxalic acid, nickel nitrate, dopamine hydrochloride powder, and tris–HCl buffer were also obtained from Sigma-Aldrich and used without further purification. KH_2_PO_4_ and Na_2_HPO_4_ were of high-grade quality from Merck (Berlin, Germany) and used as received. Distilled water and ethanol were the main solvents used throughout the work.

### 2.2. Characterization Tools

The ultraviolet-visible spectral traces of MV2B were monitored by the (PG T80+, London, UK) spectrophotometer. The FTIR spectra were recorded by JASCO FTIR-4100 (Tokyo, Japan) across the wavenumber 4000 to 200 cm^−1^. The patterns of XRD were measured using a diffractometer of GNR, APD 2000 PRO model (Tokyo, Japan). The beam of the X-ray was nickel-filtered Cu Kα (k = 1.5405 Å) radiation operated at 30 mA and 40 kV. The SEM micrographs and EDX analysis were recorded by a JSM-6510LV (JEOL, Tokyo, Japan). TEM micrographs were recorded by JEM-2100, JEOL (Tokyo, Japan). The BET measurements were made by the adsorption–desorption of N_2_ at NOVA touch 4LX (Tokyo, Japan).

### 2.3. Synthesis of Nano-NiO

Equal volumes of alcoholic solutions of oxalic acid (1 M) and nickel nitrate (0.2 M) were mixed and magnetically stirred. The solid product was filtered, washed thoroughly with a water–ethanol mixture followed with pure ethanol, left to dry in air at the ambient temperature, and then calcined for 1 h at 400 °C to obtain the nano NiO [13,23].

### 2.4. Synthesis of NiO/PDA Nanocomposite

The nanocomposite was synthesized via the addition of an appropriate volume of tris–HCl buffer (0.01 M) to 0.2 g of dopamine hydrochloride monomer in the presence of NiO (1 g). The pH of the mixture was 9.43 at the beginning of the reaction, which dropped to 8.31 after 5 h of vigorous magnetic stirring. The mixture was kept stirred overnight. The obtained brown NiO/PDA nanocomposite was washed and then vacuum dried for 24 h at 60 °C [24,28]. 

### 2.5. Removal of MV2B by Batch Mode Technique

To investigate the adsorption of MV2B on NiO/PDA, the batch mode adsorption experiments were performed. The NiO/PDA (0.05 g) was transferred to a series of Erlenmeyer flasks (100 mL), and then 18 mL of water and 2 mL of MV2B solution of the desired concentration were added. All flasks were located into a water-shaker thermostat at 120 rpm and 30 °C. At appropriate intervals of time, a flask was taken out and an aliquot sample (3 mL) of the reaction mixture was withdrawn with the aid of a Millipore filter (Cairo, Egypt) of 0.45 μm pore size to separate the particles of the composite from the medium. The concentration of the residual MV2B in reaction solution was thus determined by the use of the UV-Vis spectrophotometer (London, UK) at λ_max_ = 580 nm. The efficiency of the dye removal was determined by the equation:

(1) $$\mathrm{Efficiency}\;\%=\frac{C_{o}-C_{t}}{C_{o}}*100$$

where C_o_ is the initial concentration of MV2B in mg/L, and C_t_ is the concentration at time = t.

## 3. Results and Discussion

### 3.1. NiO/PDA Characterization

#### 3.1.1. Fourier Transform Infrared Spectroscopy (FT-IR)

**Figure 2:**
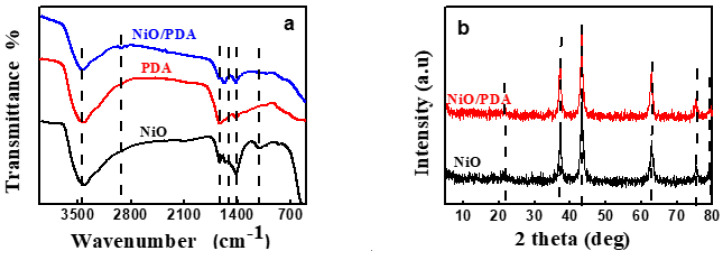
(**a**) FTIR spectra of NiO, PDA, and NiO/PDA, (**b**) XRD patterns of NiO and NiO/PDA.

FT-IR spectra were recorded in the region of 4000–200 cm^−1^. Figure 2a depicts the spectral results of NiO, PDA, and NiO/PDA. For NiO, the broad peak within 850–550 cm^−1^ is characteristic of the stretching vibration of Ni–O. The broad peak confirms the nano crystallinity of NiO. Further, the broadband at 3417.7 cm^−1^ is attributable to the stretching vibrations of O–H, while the band located at 1627.8 cm^−1^ belongs to the bending vibrations mode of H–O–H. It indicates the presence of water traces in the sample due to the adsorbed moisture. Furthermore, the peak around 1005 cm^−1^ confirms the presence of C–O and that at 1411 cm^−1^ is primarily due to the bending vibration of ionic CO_3_^2−^, while the weak band of CH_2_ is at 2911.8 cm^−1^ [13,29]. The spectrum of PDA includes peaks at 1637.3 and 1583.6 cm^−1^, which are characteristics of the aromatic ring and NH group vibrations, respectively. The 1357.2 cm^−1^ band is due to the CH_2_ bending vibration.

The peak at 1294.35 cm^−1^ is attributed to the stretching vibration of CO, while that at 3435.5 cm^−1^ can be assigned to the stretched vibration of the OH and NH groups. The relatively large band at the 1500–1100 cm^−1^ region, and the band at 1626 cm^−1^, verify the occurrence of the intramolecular cyclization reaction of dopamine along with the formation of the indole derivatives [30]. For the NiO/PDA nanocomposite, the peak at 1628.2 cm^−1^ is due to the stretching vibration of the aromatic ring. This vibration indicates that the PDA has coated the NiO nanoparticles. The peak at 2360 and 2930 cm^−1^ can be assigned to the –CH_2_ of the PDA in the nanocomposite [31].

#### 3.1.2. X-ray Diffraction Pattern (XRD)

Figure 2b shows peaks at 2θ = 37.22°, 43.035°, 62.78°, 75.37°, and 79°. These peaks refer respectively to the (111), (200), (220), (311), and (222) planes of bulk NiO. They are indexed as the face-centered cubic (FCC) of the crystalline structure of NiO because of the peak position and relative intensity. They also coincide with the standard spectrum of JCPDS, no. 04-0835. The obtained pattern of XRD indicates a single-phase sample free of any impurities, and the obtained NiO is highly pure [32].

The crystal forms of the PDA and NiO/PDA nanocomposite were also examined (Figure 2b). The pattern of NiO/PDA demonstrates the presence of the PDA loaded on NiO. The broad reflection peak at 21.53° can be assigned to the diffraction of the amorphous structures of the PDA. The five peaks of NiO/PDA at 37.22°, 43.23°, 62.97°, 75.37°, and 79.43° correspond to the diffraction planes of (111), (200), (220), (311), and (222), respectively, and indicate the presence of the FCC phase of NiO according to the JCPDS file no. 04-0783 [33].

#### 3.1.3. Scanning Electron Microscopy (SEM) and Energy Dispersive X-ray Analysis (EDX)

**Figure 3:**
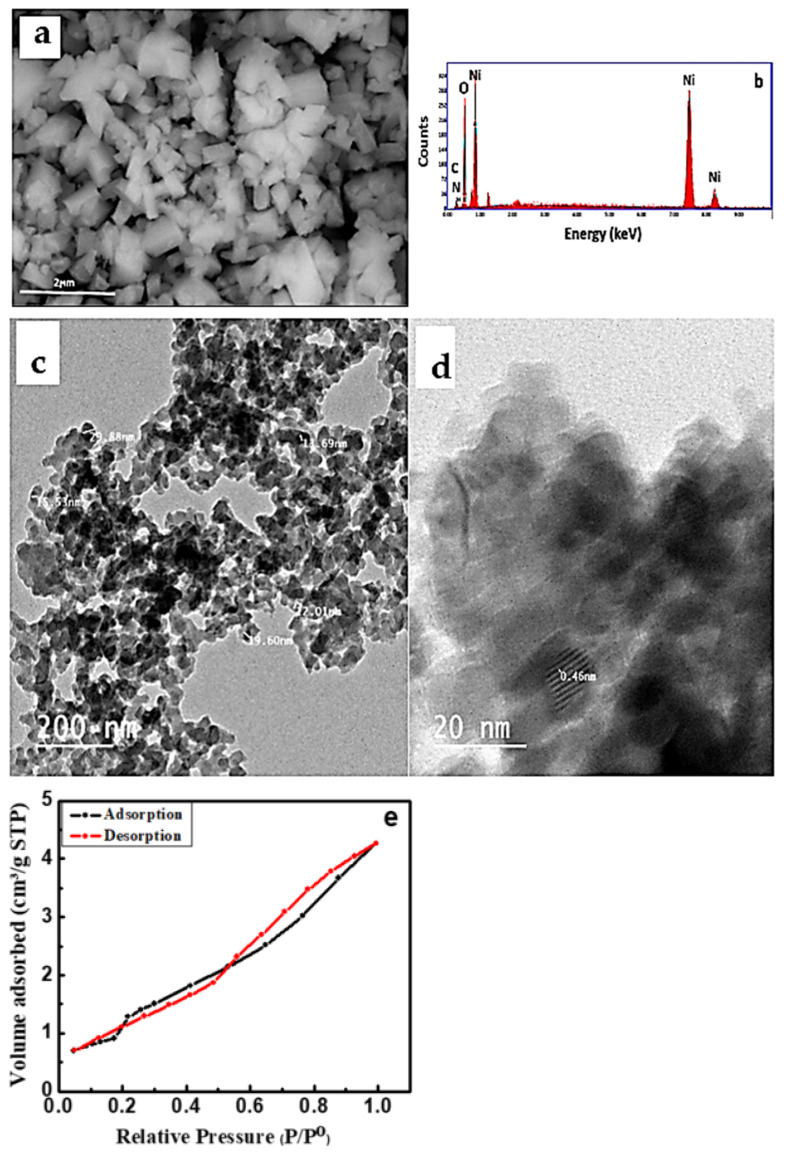
(**a**) SEM micrograph, (**b**) EDX spectra of NiO/PDA nanocomposite, (**c**) TEM (**d**) HRTEM images of NiO/PDA nanocomposite, (**e**) nitrogen adsorption/desorption isotherm for NiO/PDA nanocomposite.

The SEM micrograph of the NiO/PDA nanocomposite is presented in Figure 3a. The nanocomposite surface structure seems to be amorphous with a size of 200 nm, which is different from the spherical shape of pristine NiO [26]. The chemical analysis of the nanocomposite was determined by the EDX technique. Figure 3b illustrates the presence of Ni and O for NiO, and carbon, oxygen, and nitrogen for PDA [34]. The elemental composition percentage is provided in Table 1.

#### 3.1.4. Transmission Electron Microscopy (TEM)

Figure 3c displays the TEM micrograph of NiO/PDA. The spherical particles observed have an average particle size of 18 nm. The lattice fringes shown in HRTEM are 0.46 nm, Figure 3d. 

#### 3.1.5. Brunauer–Emmett–Teller (BET)

Figure 3e represents the N_2_ adsorption–desorption isotherm of the NiO/PDA nanocomposite. It clearly shows an IV-type isotherm with a strong hysteresis loop at a relative pressure (P/P^o^) from 0.55 to 1.0, which confirms the monodispersed ordered mesoporosity of the nanocomposite. Because of this porous structure, the surface area and the pore volume of NiO/PDA were found to be 110.591 m^2^/g and 1.4107 cm^3^/g, respectively. Such a surface area is favorable for enhancing the adsorption process. The BJH pore size distribution indicates that the average pore diameter of the NiO/PDA is 1.92233 nm [35].

### 3.2. Adsorption Efficiency of NiO/PDA

**Figure 4:**
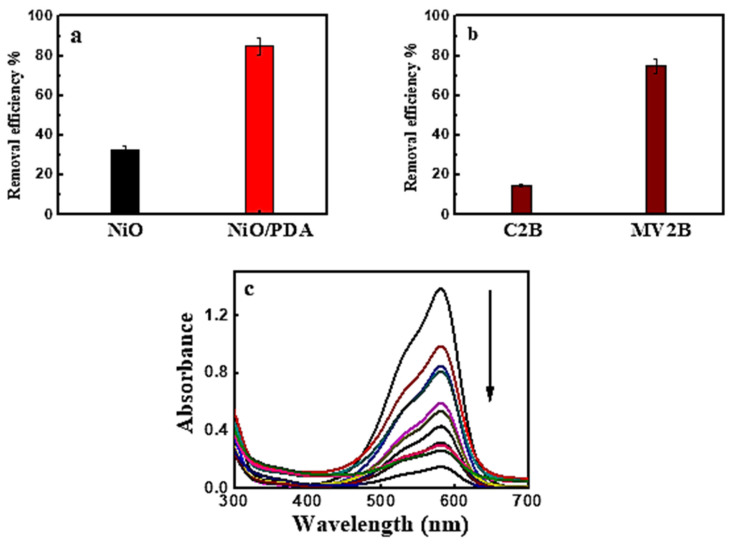
(**a**) Removal efficiency of MV2B by NiO and NiO/PDA, (**b**) removal efficiency of Chromotrope 2B and Methyl Violet 2B by NiO/PDA, NiO/PDA = 0.05 g, (MV2B)_o_ = 2.95 mg L^−1^, at 30 °C (**c**) UV-Vis spectral traces of MV2B with NiO/PDA = 0.05 g at 30 °C.

The removal percentage of MV2B, when adsorbed on NiO and NiO/PDA, is depicted in Figure 4a. The percentage was 33%, and 85%, respectively. This may be due to the successful incorporation of NiO with PDA. When NiO/PDA was used to remove the anionic Chromotrope 2B dye via adsorption, weak adsorption occurred. In contrast, strong adsorption was observed for the cationic MV2B dye, as shown in Figure 4b,c. These figures were drawn with error bars of ~5%. This value was derived from the observation during the repetitive experimental measurements of the dye adsorption under the different conditions investigated.

#### 3.2.1. Effect of NiO/PDA Dose

**Figure 5:**
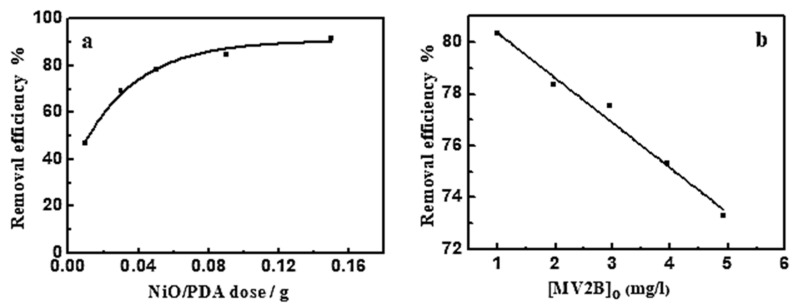
(**a**) Effect of NiO/PDA dose on the removal efficiency of MV2B. (MV2B)_o_ = 2.95 mg L^−1^, (**b**) effect of initial concentration of MV2B on its removal efficiency, NiO/PDA = 0.05 g, at 30 °C at 30 min.

Figure 5a shows the influence of NiO/PDA dose on MV2B adsorption. The adsorption efficiency increased as the dose was increased [18]. This increase comes from the increasing availability of active sites developed with the increased dose of PDA/NiO. Such an increase in the adsorption efficiency is associated with the negatively charged property of PDA in the nanocomposite, which causes preferable adsorption toward the cationic dye because of the strong catechol complexation with the amine groups with cationic dye molecules. The high ability of the NiO/PDA nanocomposite to adsorb the MV2B is due to the significant electrostatic interaction and hydrogen bonding formation between the negative charge located on the surface of PDA and the positively charged NH^+^ moiety of MV2B.

This work deals with the removal of dyes by the adsorption technique. Unfortunately, the irradiation of the two adsorbents/dye systems with the UV-Vis was not the target of this study. As the effect of lights is important in such a removal process, it can thus be taken into account in future work as photocatalytic degradation to eliminate dyes from solution.

#### 3.2.2. Effect of (MV2B)_o_

The impact of MV2B concentration on its removal efficiency was investigated at a fixed dose of NiO/PDA (0.05 g) and varying concentrations of MV2B from 0.984 to 4.92 mg L^−1^. Figure 5b provides the change in the removal efficiency as a function of MV2B concentration. This is a linear relationship between the removal efficiency % and the concentration of MV2B. The results indicate the inhibition of the adsorption process with the increasing concentration of MV2B. The inhibition is due to the gradual depletion of the vacant sites of the surface upon adsorption of the dye molecules. As long as the adsorption is continued, more and more of the active sites become occupied, ultimately leading to a decrease in the dye removal efficiency.

Various formulations of modified composites and various state-of-the-art techniques for the removal of dyes from wastewater have recently been proposed by numerous researchers [36,37,38]. Thus, new magnetic nanocomposites based on the Fe_3_O_4_ magnetite were successfully obtained by combustion and tested as adsorbents for the removal of anionic dyes such as Acid Yellow 42 and Acid Red 213 from aqueous solutions. The effect of technological parameters of solution pH, adsorbent dose, initial dye concentration, and temperature on adsorption was evaluated. The best kinetic model consistent with the experimental data was a pseudo-second order model, and the equilibrium data were correlated using the Langmuir isotherm model for the dyes under study [36]. A comprehensive study was performed to evaluate the use of an organometallic oxide chitosan-4-chloroacetophenone/CeO_2_–CuO–Fe_2_O_3_ and chitosan-4-chloroacetophenone/CeO_2_–CuO–Al_2_O_3_ nanocomposites for the removal of Red 60 disperse dye (DR) from aqueous solutions. An analysis of the molecular structure of the DR dye adsorbed on the surface of these nanocomposites showed that the adsorption process is related to the van der Waals dispersion force [37]. Recently, various physicochemical and biological wastewater treatment processes have also been studied with various types of adsorbents, such as commercial activated carbon, metal oxide adsorbents, carbon adsorbents, organometallic frameworks, and polymer adsorbents. The influence of several critical factors such as initial dye concentration, solution pH, temperature, and adsorbent dose on the dye adsorption characteristics has been investigated. In addition, the adsorption mechanisms responsible for dye removal have been elucidated on the basis of electrostatic attraction, ion exchange, surface complexation, and π–π interactions [38].

### 3.3. Adsorption Kinetics

The kinetics of the adsorption study are important for the practical application of adsorbents in removing dyes from the solution. The kinetic models are usually beneficial in elucidating the adsorption mechanism. Thus, four kinetic models were applied as described below.

#### 3.3.1. Pseudo-First-Order Kinetic Model

This model is represented as [39]:

ln (q_e_ − q_t_) = ln q_e_ − k_1_t (2)

where q_t_, q_e_, are the adsorption capacity in mg/g at specific time t, and equilibrium. k_1_ is the rate constant of adsorption. It was found that the adsorption data did not obey Equation (2), as revealed from the corresponding correlation coefficient R^2^ (0.91) derived from the plotting of ln (q_e_ − q_t_) vs. t (not provided). The data in Table 2 indicate the deviation of the current adsorption process from the first-order kinetics regime.

#### 3.3.2. Pseudo-Second-Order Kinetic Model

**Figure 6:**
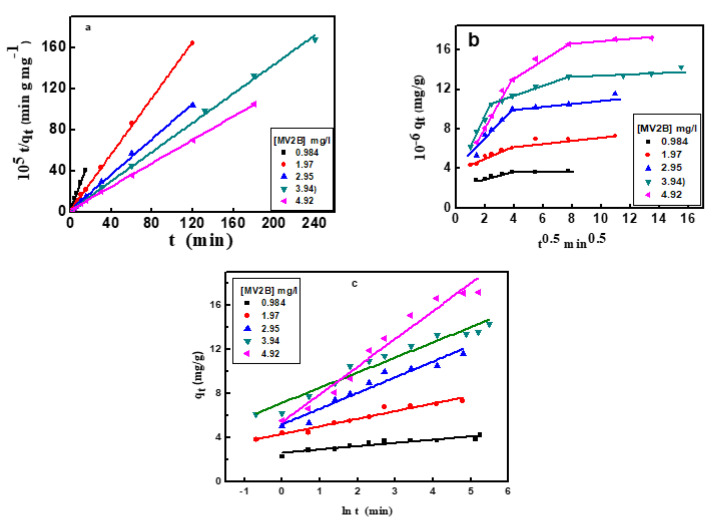
(**a**) Pseudo-second-order kinetics plots, (**b**) intraparticle diffusion model, (**c**) Elovich kinetic model for adsorption of MV2B on NiO/PDA.

The model is expressed by:

t/q_t_ = 1/k_2_q_e_^2^ + t/q_e_ (3)

where k_2_ is the pseudo-second-order rate constant (g/mg min) of interaction (Figure 6a). The derived kinetic parameters are presented in Table 2. The equilibrium adsorption amount, q_e_, of MV2B calculated from the plot is matched significantly with that observed from the experimental data. Since the R-value (0.99) is high enough for adsorption of MV2B, then NiO/PDA is perfectly commensurate with such a model. Since the rate-determining step is the share of electrons between the molecules of the dye and the NiO/PDA surface, then it suggests its chemical adsorption [40].

#### 3.3.3. Intraparticle Diffusion Model

The Weber–Morris intraparticle diffusion model was applied for the adsorption of MV2B onto NiO/PDA. It is expressed linearly by Equation (4) [39]:

q_t_ = k_p_ t^0.5^ + c (4)

where k_p_ (mg/g min^−1/2^) is the rate constant of the intraparticle diffusion. The constant c (mg/g) reflects the contribution of the boundary layer thickness into the adsorption process. A glance at Figure 6b reveals the deviation of the plot from passing through the origin point, indicating that this diffusion does not represent the rate-limiting step, and thus the adsorption of MV2B is controlled by more than one mechanism.

Generally, the greater the c-value, the higher the participation of intraparticle diffusion. Furthermore, Figure 6b exhibits linear intersecting segments for MV2B adsorption, indicating the co-existence of two or three adsorption steps. The first segment belongs to the early migration step of MV2B molecules from the liquid state to the outer surface of NiO/PDA (film diffusion). The second segment describes the diffusion of the molecules through the internal voids of nanocomposite particles [41]. At a higher concentration of MV2B, the third segment refers to the equilibrium developed from the movement of the dye molecules from the large pores to the micro-pores giving rise to a slow adsorption rate due to the saturation of the active sites on the nanocomposite surface [42]. The k_p1_ and k_p2_ values are provided in Table 2.

#### 3.3.4. Elovich Kinetic Model

The application of the Elovich kinetic model will participate in describing the kinetics of dye adsorption on the surface of NiO/PDA. It is useful and applicable for chemisorption on energetically heterogeneous solid surfaces. The model equation is:

(5) $$q_{t}=\;\frac{1}{\beta }\;\ln\;(\alpha \beta )+\frac{1}{\beta }\;\ln\;(t)$$

where α refers to the rate of the initial adsorption (mg/g min), while β denotes the desorption constant (g/mg). The q_t_ vs ln (t) plot allows for the determination of β and α from the intercept and slope (Figure 6c). The values of the kinetic constants α and β are shown in Table 2. These values changed with the change in the dye concentration [43]. As seen from Table 2, the R^2^ value is within the range 0.854–0.9611, which indicates that the adsorption process obeys the Elovich kinetic model. It was also observed that when the dye concentration increases, the R^2^ value increases, suggesting chemisorption behavior on the heterogeneous active sites of the NiO/PDA. In contrast, the desorption constant β is inversely proportional to the concentration of the dye. Hence, the reciprocal of the desorption constant (1/β) indicates an increase in the active sites on the NiO/PDA surface with increasing dye concentration, thereby reinforcing the occurrence of chemisorption.

### 3.4. Adsorption Isotherms

The adsorption of MV2B on NiO/PDA was studied following three well-known adsorption isotherm models. These models illustrate the adsorption of molecules and their distribution between the solution and the solid surface at equilibrium. The Langmuir isotherm indicates the possible formation of a monolayer from the dye molecules on the surface of NiO/PAD. The Freundlich isotherm usually refers to the heterogeneous adsorption processes, while the Dubinin–Radushkevich describes the adsorption on one type of uniform pore [44].

#### 3.4.1. Langmuir Isotherm

**Figure 7:**
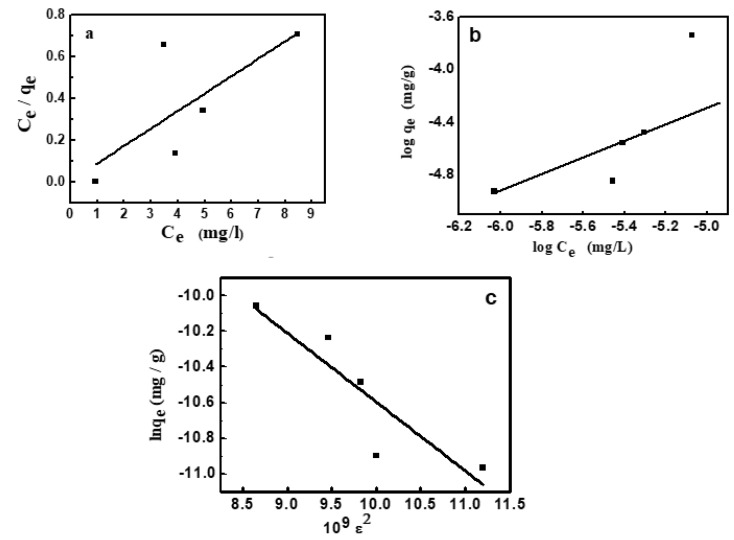
Adsorption isotherms for adsorption of MV2B onto NiO/PDA, (**a**) Langmuir, (**b**) Freundlich, (**c**) Dubinin–Radushkevich.

The model is represented by this equation:

(6) $$\frac{C_{e}}{q_{e}}=\frac{1}{K_{L}q_{m}}+\frac{C_{e}}{q_{m}}$$

where q_e_ and C_e_ are the amount of dye adsorbed and that remained in solution at equilibrium. K_L_ (1/mg) is the adsorption constant and q_m_ (mg/g) is the adsorption capacity at saturation. The values of K_L_ and q_m_ given in Table 3 [44,45] were determined from the linear plot shown in Figure 7a, whose correlation coefficient is 0.975.

A further feature of the Langmuir isotherm is the R_L_ constant, which is known as the factor of separation that is:

(7) $$R_{L}=\;\frac{1}{1+K_{L}C_{o}}$$

where the R_L_ value refers to the isotherm type and the adsorption process nature. When R_L_ is greater than 1, then the adsorption is termed unfavorable. Linear adsorption obtained at R_L_ equals 1, but in the case of 0 < R_L_ < 1, the adsorption is favorable. It becomes irreversible at R_L_ equals 0. Since the current value of R_L_ is 0.0453, it thus confirms the favorable adsorption of MV2B on the present NiO/PDA nanocomposite.

#### 3.4.2. Freundlich Isotherm

The isotherm assumed heterogeneous adsorption owing to the variation of adsorption sites. This isotherm is featured with multilayer adsorption and the non-linear distribution of energy across the adsorption sites [46]. It has the main constants K_F_ and 1/n, which were determined from the linear plot between log q_e_ and log C_e_ according to Equation (8) and given in Figure 7b:

(8) $$\log\;q_{e}=\log\;K_{F}+\frac{1}{n\;}\;\log\;C_{e}$$

K_F_ and n refer respectively to the capacity of adsorption and its strength (Table 3). From the value of 1/n, the favorable adsorption of MV2B on the NiO/PDA and the surface heterogeneity degree can be quantified. When 1/n is < 1, then favorable adsorption occurs and its capacity increases with the generation of new active sites for adsorption on the surface [47]. Furthermore, when n is less than unity, the adsorption is chemical; greater than unity, the adsorption is physical [48]. In the present study, 1/n = 1.069 and n = 0.934, indicating the favorable chemical adsorption of MV2B on the surface of NiO/PDA.

#### 3.4.3. Dubinin–Radushkevich (D–R) Isotherm

The application of this isotherm distinguishes whether the MV2B adsorption on NiO/PDA is physical or chemical. The model is characterized by Equation (9) [49]:

lnq_e_ = ln q_m_ − β ε^2^ (9)

where ε is the Polanyi potential, q_m_ is the capacity of a monolayer formation (mg/g), β relates to the average energy of adsorption, and ε can be determined by Equation (10):

(10) $$\varepsilon =\mathrm{RTln}\;(1+\frac{1}{C_{e}})$$

where q_m_ and β were obtained from the linear plot drawn between lnq_e_ and ε^2^, which is given in Figure 7c. The energy (E) of dye adsorption was obtained from Equation (11):

(11) $$E=\frac{1}{\sqrt{-2\beta }}$$

The obtained value of E is 35.96 kJmol^−1^. It confirms that MV2B was chemically adsorbed on NiO/PDA, since the obtained value of E is greater than 16 kJmol^−1^. If 1 < E > 16, the adsorption is physical [50].

### 3.5. Effect of pH

**Figure 8:**
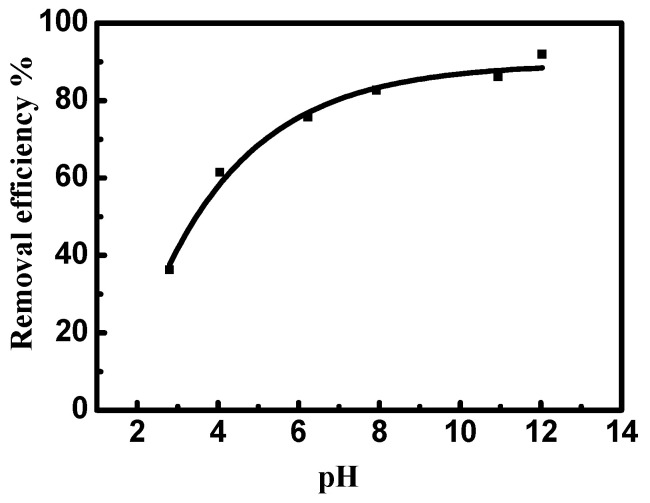
Effect of pH on the removal efficiency of MV2B by NiO/PDA. (MV2B)_o_ = 2.95 mg L, NiO/PDA = 0.05 g, at 30 °C.

Figure 8 illustrates the influence of pH on the removal of MV2B by NiO/PDA. It can be observed that MV2B removal by NiO/PDA is largely dependent on the solution pH. The removal percentage increases gradually with the increase of pH. The dependence of adsorption on the pH can well be interpreted by the surface charges of NiO/PDA. At low pH, the positive charge of NiO/PDA surface generated by the formation of the NH_3_^+^ or OH_2_^+^ species will cause a strong electrostatic repulsion with the positively charged MV2B molecules in solution, resulting in a drop in the efficiency of the dye removal. In contrast, at the alkaline range of pH, the NiO/PDA acquires negative charges due to the adsorbed OH^−^ moieties on its surface and also the phenolic group deprotonation of the PDA in NiO/PDA. The negatively charged surface of the NiO/PDA will thus produce a strong electrostatic attraction toward the MV2B molecules and, consequently, the removal process increases. Moreover, the existence of active amine or catechol components inside the NiO/PDA can also further improve the MV2B capture via electrostatic attraction [51,52].

### 3.6. Reusability

**Figure 9:**
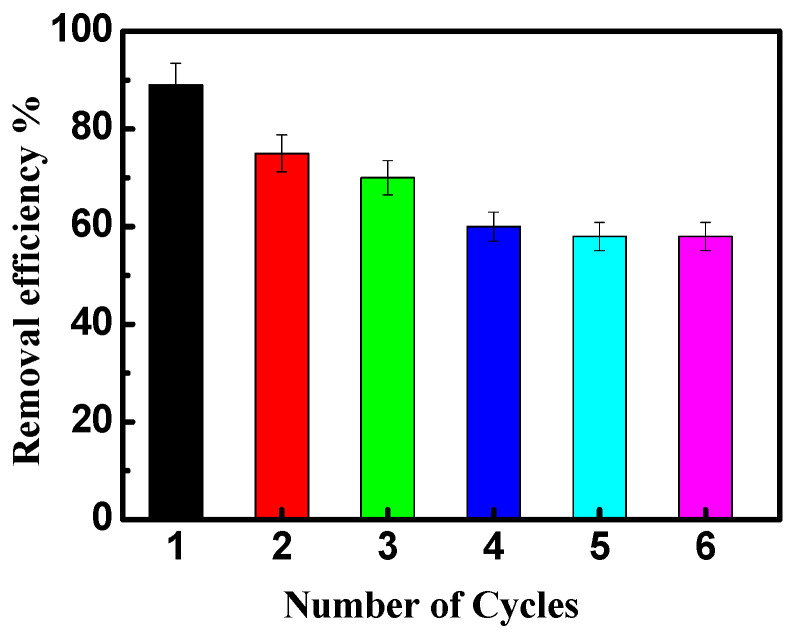
The reusability of NiO/PDA during adsorption of MV2B.

The reusability of NiO/PDA is presented in Figure 9. After the adsorption run was completed, the nanocomposite was regenerated for further reuse experiments. The exhausted NiO/PDA was readily regenerated by rinsing it in H_2_O_2_ solution at room temperature. It is clear from Figure 9 that the adsorption efficiency of the dye decreased from 88 to 58% after six cycles, indicating the good stability of the nanocomposite. This loss of efficiency may be due to the possible degradation of polydopamine during the regeneration of the nanocomposite with H_2_O_2_ via Fenton oxidation [51,53]. Therefore, this experiment has proven that NiO/PDA can be used as a recyclable adsorbent.

It is a good idea to carry out a comparative analysis of the results obtained in the framework of this study with existing and previously obtained results. However, there are a number of insurmountable obstacles to this. The approaches adopted in this work are not used in all similar previous studies. The approaches are seen in some, but not all published works. To establish a comparable table summarizing the results of the current work with that existing in the literature, all optimal conditions, such as the type of dye and its concentration, adsorbent and its dose, pH, and temperature, must be similar except for one variable. This is very difficult, but can be possible. This can only be done if the target of the research is originally planned to fulfill such a comparison under controllable conditions. Again, this can be an interesting approach for future research.

## 4. Conclusions

NiO was modified with PDA to give the NiO/PDA nanocomposite. The major elements found were C, N, O, and Ni, indicating the successful impregnation of the PDA into the NiO. The average radius of the nanocomposite particles was in the range of 13.69–29.88 nm. The specific surface area was 110.591 m^2^/g and the pore volume was 1.4107 cm^3^/g. The dye removal efficiency by this nanocomposite was higher than that of the parent NiO. The nanocomposite was proven to be a highly active adsorbent for the cationic MV2B, and less active toward the anionic Chromotrope 2B. The removal efficiency was dependent on the adsorbent dose, initial concentration of the dye, and the pH. The adsorption process followed the pseudo-second-order, intraparticle diffusion, and Elovich kinetic models and obeyed the Langmuir isotherm. The optimum conditions of the process were (MV2B)_o_ = 0.984 mg/L, 0.15 g of NiO/PDA, and pH 12. The nanocomposite was stable, so it can be reused for several cycles without a high loss of activity. Based on these results, the removal of MV2B was found to be a chemisorption process, and thus it can be tuned for application in the treatment of wastewater from other pollutant dyes. 

## Figures and Tables

**Table 1:** EDX analysis of NiO/PDA nanocomposite.

Elements	Weight %
C	8.62
N	0.81
O	18.74
Ni	71.83

**Table 2:** Adsorption kinetics models for the adsorption of MV2B onto NiO/PDA (0.05 g) at 30 °C.

(MV2B)_o_ mg/L	Pseudo-First-Order Model				Pseudo-Second-Order Model				Intraparticle Diffusion Model					Elovich	
	q_e.cal_ mgg^−1^	q_e.exp_ mglg^−1^	k_1_ min^−1^	R^2^	q_e.cal_ mgg^−1^	q_e.exp_ mgg^−1^	k_2_ g mg^−1^ min^−1^	R^2^	k_p1_ mg g^−1^ min^−1^	R^2^	k_p2_ mg g^−1^ min^−1/2^	R^2^	α mg g^−1^ min^−1^	Β g.mg^−1^	R^2^
0.984	0.212	1.799	4.7262	0.7255	0.212	0.3808	0.6425	0.9982	0.3546	0.9584	0.0153	0.980	9.25	3.3823	0.854
1.972	0.747	3.878	1.0423	0.9103	0.747	0.7342	0.2534	0.9994	0.6285	0.9324	0.1758	0.900	81.65	1.4502	0.947
2.953	1.243	2.223	0.0027	0.8399	1.243	1.1763	0.5626	0.9979	1.7636	0.9279	0.1293	0.999	216.32	0.7293	0.954
3.942	1.434	5.482	0.0133	0.6675	1.434	1.4142	1.0053	0.9985	2.8335	0.9819	0.5317	0.990	715.31	0.7017	0.934
4.923	1.716	1.433	0.4322	0.8638	1.716	1.7623	1.2877	0.9996	2.7012	0.9812	0.9117	0.925	1354.92	0.3974	0.961

**Table 3:** Adsorption isotherm constants for adsorption of MV2B on NiO/PDA (0.05 g). (MV2B)_0_ = 2.95 mg L^−1^ at 30 °C.

Isotherm	Parameters	Value
Langmuir	q_m_	12. 04 mgg^−1^
	K_L_	7.1347 Lmg^−1^
	R^2^	0.9750
	R_L_	0.0453
Freundlich	K_F_	2.4434 Lmg^−1^
	1/n	1.3044
	N	0.767
	R^2^	0.9567
Dubinin–Radushkevich	E	35.958 kJmol^−1^
	R^2^	0.890

## Data Availability

Not applicable.
